# Triple metachronous primary pancreatic and gallbladder cancer associated with pancreaticobiliary maljunction: a case report

**DOI:** 10.1186/s40792-021-01160-4

**Published:** 2021-04-06

**Authors:** Yuta Kuhara, Yasushi Hashimoto, Satoshi Hirahara, Haruna Kubota, Kenji Shirakawa, Kazuhiro Toyota, Raita Yano, Hironori Kobayashi, Yujiro Yokoyama, Yoshihiro Sakashita, Yoshiaki Murakami, Kiyomi Taniyama, Katsunari Miyamoto

**Affiliations:** 1 Department of Surgery, Hiroshima Memorial Hospital, Honkawa-cho1-4-3, Naka-ku, Hiroshima, 730-0802 Japan; 2 Department of Pathology, Hiroshima Memorial Hospital, Hiroshima, Japan

**Keywords:** Pancreaticobiliary maljunction, Gallbladder cancer, Pancreatic cancer, Remnant pancreatic cancer, Metachronous cancers, Triple cancers

## Abstract

**Background:**

Metachronous pancreatic and gallbladder cancer is a rare condition and has a dismal prognosis. Herein, we present a patient with triple metachronous primary pancreatic and gallbladder cancer associated with pancreaticobiliary maljunction who achieved long-term survival after undergoing repeat curative surgery.

**Case presentation:**

A 65-year-old female patient with advanced gallbladder cancer associated with pancreaticobiliary maljunction underwent extended cholecystectomy with extrahepatic bile duct resection. The pathological diagnosis was T3N0M0 stage III A papillary adenocarcinoma with hepatic invasion. During a monthly follow-up, a diffuse hypovascular 2.0 × 1.5-cm mass was detected in the pancreatic head 6.2 years after the initial surgery. Hence, the patient underwent pancreaticoduodenectomy. Histological examination revealed T3N0M0 stage IIA well-differentiated adenocarcinoma without lymph node metastases. Marked inflammatory reaction was observed in the non-cancerous lesions of the proximal pancreatic head parenchyma containing bile pigment within ductular lumens. After 12.5 years from the initial surgery, total pancreatectomy for a 4.0 × 3.0-cm mass in the remnant pancreas was performed. Histological examination revealed T3N1M0 stage IIB moderately differentiated adenocarcinoma with lymph node metastases. Hence, surgical curative resection was achieved. Based on the pathological findings, a definitive diagnosis of triple metachronous pancreatic and gallbladder cancer was made. The pathology suggests no precursor lesions such as pancreatic intraepithelial neoplasia (PanIN) and atypical flat lesions, but marked inflammations in the non-cancerous lesions, strengthening our hypothesis that chronic inflammation induced by the pancreaticobiliary maljunction is related to carcinogenesis of the pancreas. Despite further adjuvant chemotherapy, the patient’s general condition worsened; however, she remained alive 15.2 years after the initial surgery while receiving the best supportive care.

**Conclusions:**

Repeat curative surgery for triple metachronous cancer was associated with a favorable prognosis. Both the biliary tract and the pancreas should be closely monitored during follow-up among patients with pancreaticobiliary maljunction, which can be managed with curative surgery.

## Background

Metachronous pancreatic and gallbladder cancer is a rare condition and has a dismal prognosis [1]. Patients with pancreaticobiliary maljunction are at high risk of developing biliary tract cancer and pancreatitis [2]. Few cases of multifocal cancer associated with this anomaly have been reported [1]. However, the etiology of pancreatic cancer in individuals with pancreaticobiliary maljunction remains unclear [2]. Herein, we report a rare case of triple metachronous primary pancreatic and gallbladder cancer associated with pancreaticobiliary maljunction in a patient who achieved long-term survival after undergoing repeat curative surgery.

## Case presentation

**Fig. 1:**
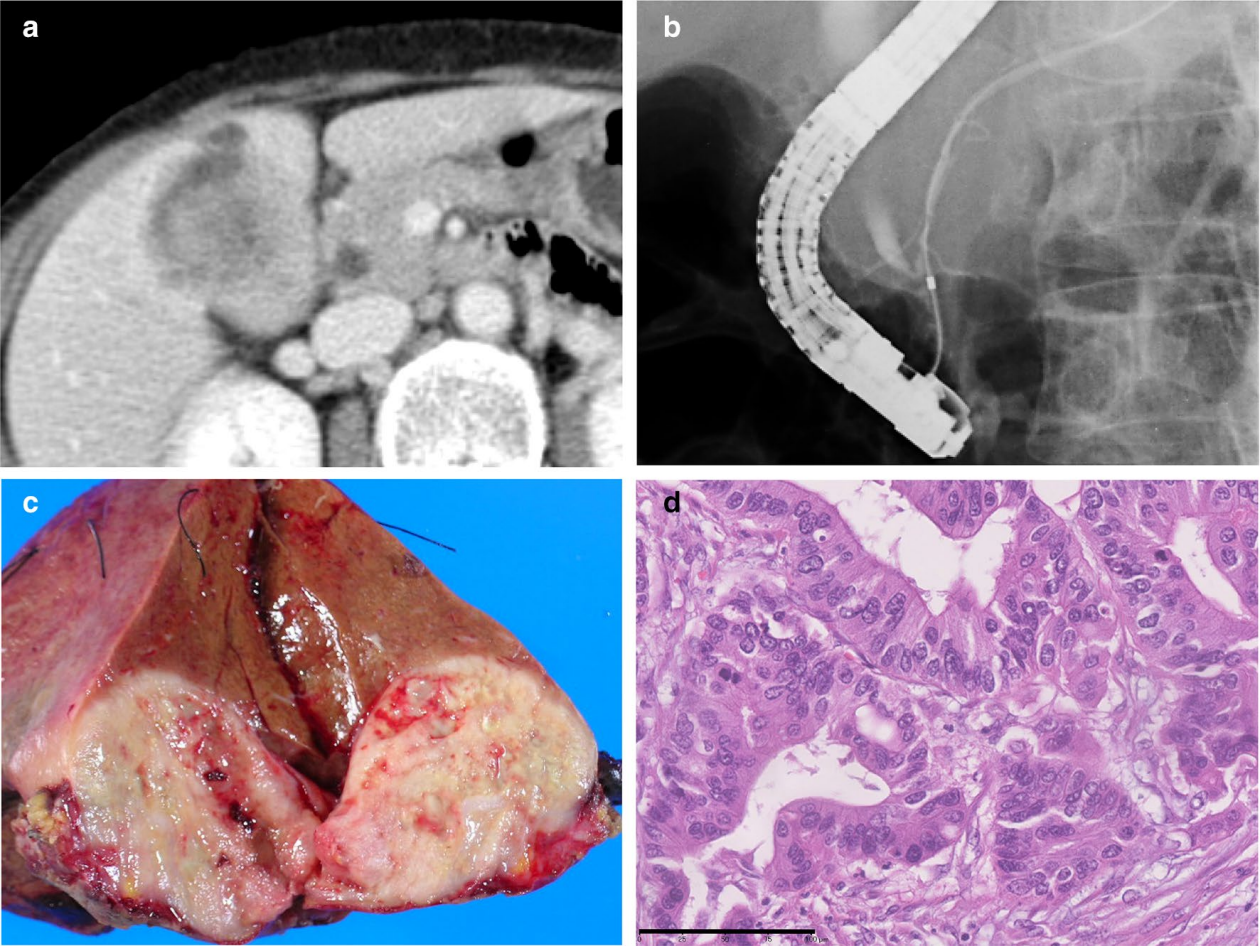
**a** Computed tomography scan showed a hypovascular irregular 3.5 × 3.0 cm mass in the gallbladder infiltrating to the adjacent hepatic parenchyma. **b** Endoscopic retrograde cholangiopancreatography showing the communication between pancreatic and bile ducts maintained despite contraction of the sphincter, which revealed pancreaticobiliary maljunction with biliary dilatation. **c** Macroscopic photo of the resected specimens of the gallbladder carcinoma. **d** Histologically, papillary adenocarcinoma of the gallbladder invaded to the adjacent liver parenchyma (bar indicates 100 µm)

**Fig. 2:**
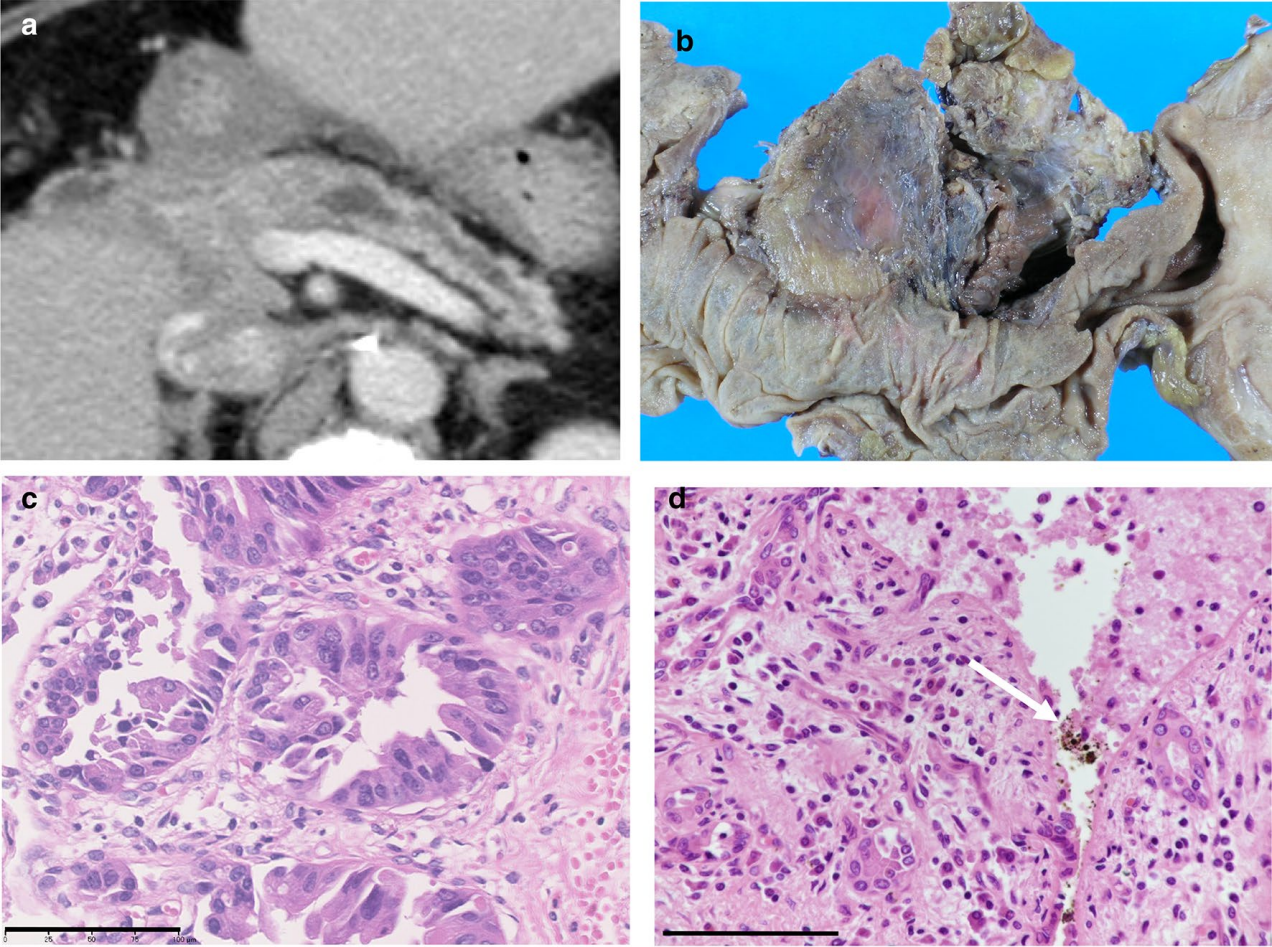
**a** Computed tomography scan showing a diffuse and low-density 2.0 × 1.5 cm mass in the pancreatic head after 6.2 years from the initial surgery. The distal pancreatic duct was dilated, and there was evidence of associated pancreatitis. **b** Macroscopic photo of the resected specimens of the pancreatic head carcinoma. **c** Histologically, tumor consisted of well-differentiated ductal adenocarcinoma which resembles pathological appearances to the gallbladder carcinoma (bar indicates 100 µm). **d** Distinctive features of non-cancerous pancreatic parenchyma. Marked inflammatory reaction containing bile pigment within ductular lumens (arrow) in the proximal non-cancerous lesions (bar indicates 100 µm)

**Fig. 3:**
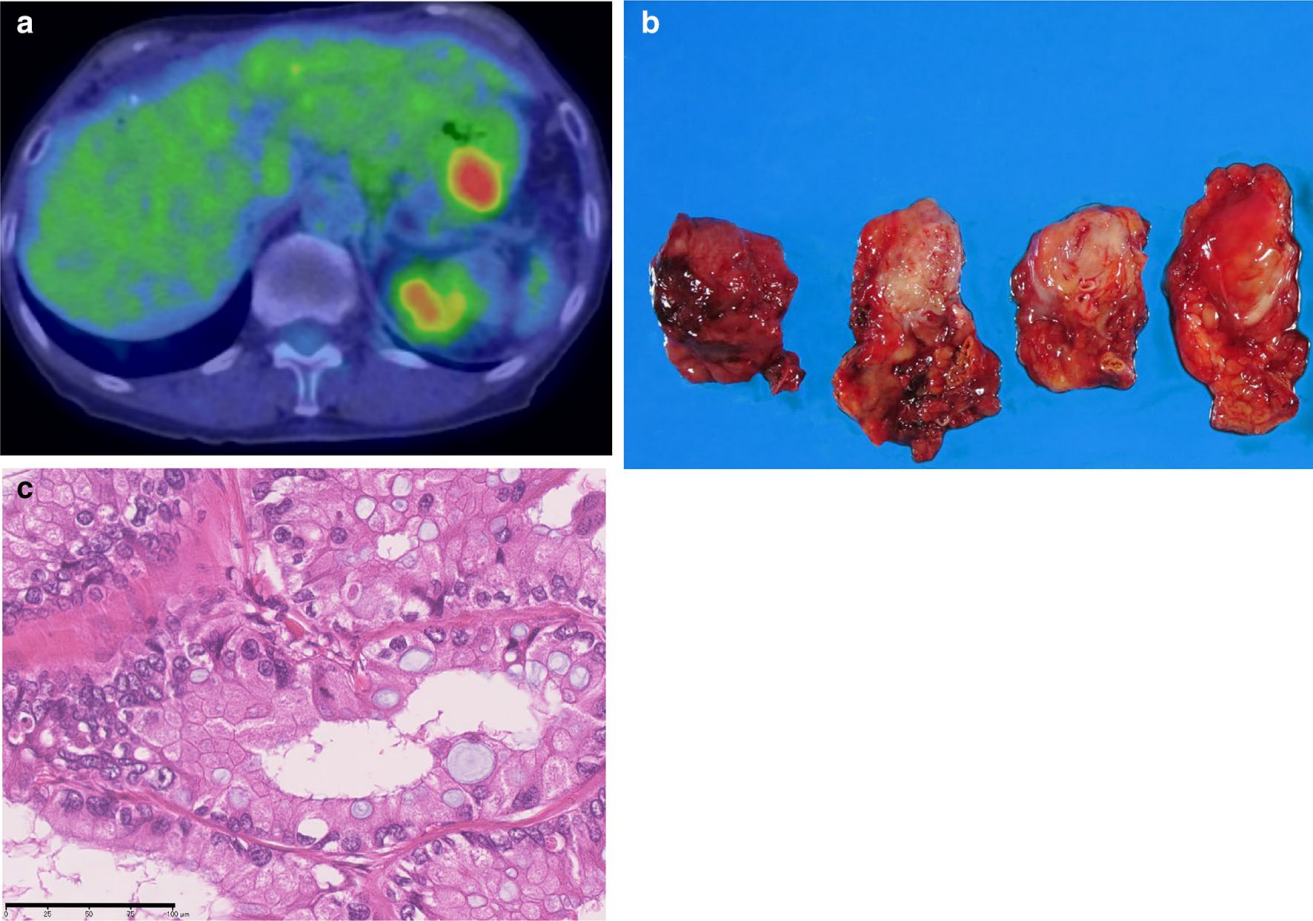
**a** Positron emission tomography/computed tomography using 18F-fluorodeoxyglucose showed hypermetabolism with a maximum standardized uptake value of 6.2 after 12.5 years from the initial surgery. **b** Macroscopic photo of the resected specimens of the remnant pancreatic carcinoma. **c** Histology revealed moderately differentiated ductal adenocarcinoma in the tail of the pancreas apart from the anastomotic site (bar indicates 100 µm)

A 65-year-old female patient was referred to our hospital due to right hypochondralgia and weight loss. Computed tomography (CT) scan revealed a 3.5 × 3.0 cm mass in the gallbladder that invaded the adjacent hepatic parenchyma. Further, pancreaticobiliary maljunction was confirmed via direct cholangiography (Fig. 1a, b). She underwent extended cholecystectomy with extrahepatic bile duct resection, and curative resection was achieved. The pathological diagnosis was T3N0M0 stage IIIA (TNM classification) papillary adenocarcinoma of the gallbladder with hepatic invasion (Fig. 1c, d). No adjuvant treatments were provided. Under our hospital policy, the patient was under continuous follow-up of functional outcomes and oncological surveillance for over 5 years. A diffuse hypovascular 2.0 × 1.5 cm mass in the pancreatic head was detected 6.2 years after the initial surgery (Fig. 2a). Hence, pancreaticoduodenectomy was performed. Histological examination revealed T3N0M0 stage IIA well-differentiated adenocarcinoma infiltrating into the posterior tissue of the pancreatic head with R0 resection (Fig. 2b, c). Marked acute and chronic inflammation was observed in the non-cancerous lesions of the pancreatic head parenchyma containing bile pigment within ductular lumens (Fig. 2d). The patient further received adjuvant chemotherapy with gemcitabine for 12 months. After 12.5 years from the initial surgery, a 4.0 × 3.0 cm mass in the remnant pancreatic tail apart from the anastomotic site was detected (Fig. 3a). The patient subsequently underwent total pancreatectomy, and surgical curative resection was achieved. Histological examination revealed T3N1M0 stage IIB moderately differentiated adenocarcinoma with lymph node metastases (Fig. 3b, c). Based on these findings, a definitive pathological diagnosis of metachronous triple adenocarcinomas in the pancreaticobiliary tract was made. The pathology suggests no precursor lesions such as PanIN and atypical flat lesions in the pancreatic specimens, but marked inflammatory change was observed in association with exocrine pancreatic gland loss in the non-cancerous pancreatic parenchyma. The patient received adjuvant chemotherapy with gemcitabine for another 6 months. Then, she developed multiple liver metastases, but remained alive 15.2 years after the initial surgery while receiving the best supportive care.

## Discussion

Metachronous malignancies are defined as two or more primary cancers diagnosed after more than 6 months from the initial primary cancer [3]. There are several case reports about metachronous cancers of the brain, breast, and urogenital and gastrointestinal tract [4–6]. However, no cases of triple metachronous primary pancreatic and gallbladder cancer have been reported.

Pancreaticobiliary maljunction is a congenital anomaly in which the junction between the pancreatic duct and the common bile duct is outside the sphincter of Oddi. The overall incidence of biliary cancers is 200 times higher in patients with pancreaticobiliary maljunction than in the general Japanese population [7]. In such a case, biliopancreatic and pancreaticobiliary refluxes occur, resulting in various pathological conditions in the biliary tract and the pancreas. The biliopancreatic reflux specifically activates pancreatic enzymes in the pancreatic ducts, which may cause chronic inflammation and metaplastic epithelial change in the pancreas [2]. The etiology of pancreatic cancer in this anomaly remains unclear. We hypothesize that acute and chronic inflammatory change induced by biliopancreatic reflux may eventually result in cancer development of the pancreas as we observed in biliary cancers in the pancreatobiliary maljunction patients. In our case, the pathology suggests no precursor lesions such as PanIN and atypical flat lesions, but there are evidence of marked inflammatory changes containing bile pigment within ductular lumens in the proximal non-cancerous lesions of the pancreas, which we believe strengthens our hypothesis.

The postoperative 5-year survival rate of metachronous pancreatic cancer after pancreatic resection is approximately 16–46%, and recurrence develops generally within 2 years [8, 9]. The mainstream treatment for recurrent pancreatic and gallbladder cancer is chemotherapy or chemo-radiotherapy. However, recent clinical investigations have shown that local resection may have an oncologic role in recurrent pancreatic cancer. A project study about pancreatic surgery was conducted by the Japanese Society of Hepato-Biliary-Pancreatic Surgery. Results showed that repeat pancreatectomy for remnant pancreatic cancer had a favorable outcome. That is, the median survival time of the patients was 26 months, and that of the non-resected group was 14 months (hazard ratio: 0.56; *P* = 0.012) [8]. In the study of Miyasaki et al., patients who underwent repeat pancreatectomy had a favorable prognosis. That is, the 2- and 5-year survival probability rates were 61% and 46% in the resected group and 19% and 6.2% in the non-resected group, respectively (*P* < 0.01) [9]. The current case provides a different perspective for patients with pancreaticobiliary malfunction in terms of the chemical pathway in pancreatic cancer. Further, long-term follow-up protocols were effective.

## Conclusions

Herein, we present a rare case of triple metachronous pancreatic and gallbladder cancer associated with pancreaticobiliary maljunction. The patient achieved a 15-year recurrence-free survival after the initial resection. The association between pancreatic carcinoma and pancreaticobiliary maljunction remains unclear. However, both the pancreas and the biliary tract should be closely monitored among patients with this anomaly, which can be treated with curative resection and is associated with long-term survival.

## Data Availability

All data generated or analyzed during this study are included in this published article.

## Abbreviations

PanIN: Pancreatic intraepithelial neoplasia
CT: Computed tomography
